# A dynamical model of perceptual categorization

**DOI:** 10.1186/1471-2202-12-S1-P265

**Published:** 2011-07-18

**Authors:** Daniel Martí, John Rinzel

**Affiliations:** 1 Center for Neural Science, New York University, New York, NY 10003, USA; 2 Courant Institute of Mathematical Sciences, New York University, New York, NY 10012, USA

## 

In visual and auditory scenes, we are able to identify shared features among sensory objects and group them according to their similarity. We consider the case of temporally streamed input: sequentially presented or sampled objects, disregarding (in the case of vision) spatial arrangement. The process of grouping by similarity is easy, fast, and does not require attention or training when the stimuli are sufficiently simple. What neural mechanisms govern such a grouping process remains an open question. We propose a high-level, neuro-mechanistic model of perceptual grouping based on the framework of continuous attractor networks [1,2]. The network model is able to dynamically cluster the features of the sensory objects present in the input stream, thereby performing an elementary categorization of the sensory scene. The resulting categories are represented by self-sustained selective patterns of neuronal activity, or “bumps”, in which only a fraction of neurons with similar selectivity properties are active. Such activity patterns can be thought of as localized in feature space, and persist in the absence of inputs due to recurrent excitation. The connectivity of the network is sufficiently structured for these non-homogeneous patterns to be stable, while is mild enough to maintain the stability of a homogeneous, low-activity state, associated with spontaneous activity. The bistability between the homogeneous and the localized states allows for the formation of categories conditioned on the statistics of the external inputs, idealized as temporal sequences of single feature values. We suppose that the presentation of a single item in the sequence elicits a subthreshold response that, compared to the stimulus presentation rate, decays slowly after the stimulus is removed. Because of the slow decay, the network responds to the sequence of stimuli by effectively summing temporally the responses triggered separately by each item. Only when the input stream consists of an adequate number of similar feature values, will there be successive hits within a critical time window in one or more localized regions of feature space that will be sufficient to trigger the emergence of one or more bump states. Each such bump state encodes approximately the mean feature value of the items belonging to a particular category, and hence can be regarded as the neural representation of a category prototype. Using a combination of analytical and numerical methods, we study the conditions for the network to be capable of forming categories. More specifically, we investigate the necessary conditions that the connectivity has to satisfy so that multiple localized states can coexist, while preserving the bistability with a homogeneous state. These conditions can be fulfilled when the connectivity has a narrow Mexican-hat profile, and there is an approximate balance between excitation and inhibition. A generalization of the model for multi-dimensional features is also analyzed and leads to similar results. We characterize the ability of the network to extract clusters of features as a function of the statistics of the input stream, and show that results are consistent with the psychophysics of perceptual categorization and ensemble statistics [3].
